# SiC transmission-type polarization rotator using a large magneto-optical effect boosted and stabilized by dressed photons

**DOI:** 10.1038/s41598-020-69971-3

**Published:** 2020-07-31

**Authors:** Takuya Kadowaki, Tadashi Kawazoe, Motoichi Ohtsu

**Affiliations:** 1 Nichia Corporation, 3-13-19 Moriya-cho, Kanagawa-ku, Yokohama, Kanagawa 221-0022 Japan; 2 Tokyo Denki University, 5 Senju-Asahi-cho, Adachi-ku, Tokyo, 120-8551 Japan; 3 Research Origin for Dressed Photon, 3-13-19 Moriya-cho, Kanagawa-ku, Yokohama, Kanagawa 221-0022 Japan

**Keywords:** Nanoscience and technology, Optics and photonics

## Abstract

This paper reports the fabrication and operation of a transmission-type polarization rotator for visible light with a wavelength of 450 nm using indirect-transition-type semiconductor crystalline SiC in which Al atoms were implanted as a p-type dopant. A novel dressed-photon–phonon (DPP)-assisted annealing method was used for fabrication. The fabricated device exhibited a gigantic magneto-optical effect induced by interactions between photons, electrons, phonons, and magnetic fields in a nanometric space, mediated by dressed photons. The optical path length for polarization rotation was as short as the thickness of the p–n junction. It operated with a weak magnetic field on the order of mT, generated by injecting current to a ring-shaped electrode on the device surface. The Verdet constant was as large as 9.51 × 10^4^ rad/T.m at a wavelength of 450 nm. SQUID measurements confirmed that the SiC crystal exhibited conspicuous ferromagnetic characteristics as a result of the DPP-assisted annealing. In this device, the dressed photons boosted the magnitude of the magneto-optical effect and stabilized the device operation of the polarization rotator.

## Introduction

Owing to recent progress in optical information processing technology, the demand for high-performance functional optical devices is increasing. Among such devices, a magneto-optical spatial light modulator and optical isolator using the magneto-optical effect are the most promising devices because they play essential roles in the control and processing of optical information^1–3^. However, since these devices have been conventionally fabricated using ferromagnetic materials such as yttrium iron garnet (YIG), one major problem is their large optical absorption in the visible range, originating from Fe atoms in the crystal^4^. On the other hand, even though this absorption is low in the case of optical isolators made of transparent magneto-optical materials such as terbium gallium garnet (TGG), the problem is their low polarization rotation capabilities^5^, resulting in the need for a long optical path length to achieve the desired rotation. These technical situations indicate that it is not straightforward to find novel materials having both low absorption and high polarization rotation capability in the visible range. An additional problem is that an external coil or a bulky magnet is required to generate a strong magnetic field for the device operation.

In order to solve these problems, a visible reflection-type polarization rotator was developed by using a wide-bandgap ZnO semiconductor by employing fabrication and operation methods based on quite different principles from those used conventionally^6^. Even though ZnO is a semiconductor that does not exhibit any ferromagnetic characteristics, large polarization rotation was demonstrated by this device. This was attributed to the interaction between photons, electrons, phonons, and current-induced magnetic fields mediated by spatially localized dressed photons (DPs) created in the regions around dopants in a nanometric space (a detailed discussion of DPs is given in the “Basics” section below). However, the crystallographic properties of ZnO are not yet sufficiently high for this material to be used for advanced optical devices, even though it is transparent in the visible range. Also, it is not straightforward to grow p-type ZnO substrates even by using a recent sophisticated doping method^7^.

On the other hand, since the wide bandgap semiconductor SiC has a high breakdown electric field strength, and p-type substrates have been grown by using recent advanced technology, it has been advantageously applied to novel power electronic devices^8–10^. Due to the technical trends in these applications, it is expected that transparent SiC crystals exhibiting high crystallographic properties could be supplied in a sustainable manner from now on. By noting this advantage and expectation, basic experimental results on a reflection-type SiC polarization rotator for visible light with a wavelength of 450 nm have been recently reported by two authors (T.K. and M.O.) of this paper^11^. The fabrication and operation principles of this device are based on the concept of the DP and are equivalent to those of highly efficient silicon light-emitting devices (i.e., LEDs and LDs)^12–18^,specifically, these Si devices emit photons with high efficiency by exchanging the momenta of the electrons in the conduction band with those of phonons, which are the constituent elements of the DPs.

This paper reports the fabrication and operation of a novel *transmission-type* polarization rotator for a wavelength of 450 nm using a single-crystal SiC.

## Basics

The DP had previously been called an optical near field from the viewpoint of classical wave optics. However, as a result of more recent careful theoretical and experimental studies on light-matter interactions in a nanometric space based on quantum field theory, it was renamed as the DP due to its unique quantum optical nature. The detailed nature of the DP has been described in^19^. In summary, these studies indicate that the DP is a quantum field created as a result of the interaction between photons and electrons in a nanometric space. They have shown that the created DP could excite multi-mode coherent phonons in the crystal and couple with them, resulting in the creation of a novel quantum field called a dressed-photon–phonon (DPP)^20^. For device fabrication and operation, DPPs must be created efficiently in the semiconductor crystal. They can be created by autonomously controlling the spatial distribution of dopant atoms in the crystal using a DPP-assisted annealing method. In the annealing, Joule energy is generated by injecting a current into the p–n junction. Upon being heated by this Joule energy, the dopant atoms randomly diffuse in the crystal. During the heating, the crystal surface is irradiated with light that propagates through the crystal without absorption when its photon energy is lower than the bandgap energy of the crystal. As a result, the irradiated photons couple with electrons in a nanometric space at the dopant atoms to create DPs. Furthermore, the created DPs couple with multi-mode coherent phonons in the crystal, resulting in DPP creation.

Due to momentum exchange with phonons in the DPPs, the electrons in the conduction band recombine with positive holes to create photons, which is a stimulated emission process triggered by the irradiated light. Thus, the local diffusion efficiency of the dopant atoms decreases because a part of the Joule energy for heating is converted into the propagating photon energy and dissipates out from the crystal. By continuing the heating and dissipation processes above, the spatial distribution of the dopant atoms varies autonomously and gradually reaches a stationary state that is optimized for creating DPPs most efficiently. As a result, the device becomes optically active, allowing emission of light and/or rotation of the polarization of the incident light very effectively. When operating the device fabricated by using a SiC crystal, the DPPs are efficiently created at the optimally distributed dopant atoms by the incident light, and thus, the device exhibits a gigantic magneto-optical effect even though SiC is an indirect-transition-type semiconductor, and the polarization of the incident light is efficiently rotated. Detailed theoretical descriptions of the DPP-assisted annealing method and experimental results have been reported in previous papers by the research groups of some of the authors^21,22^.

## Experiment

**Figure 1:**
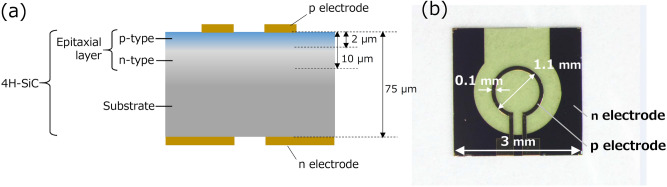
Profile of a polarization rotator using a 4H-SiC crystal. (**a**) Cross-sectional structure. (**b**) Optical microscope image.

Figure 1a schematically explains the cross-sectional structure of the SiC substrate. The (0001) planar surface of a 4H-SiC crystal was ion-implanted with a p-type dopant (Al atoms). After a ring-shaped electrode (outer diameter: 1.1 mm, line width: 0.1 mm) and a planar electrode were deposited on the top and bottom surfaces, respectively, the substrate was diced to form a 3 mm × 3 mm square device, as shown by the optical microscope image in Fig. 1b. In order to create the DPPs efficiently for fabrication and operation of the SiC device, the DPP-assisted annealing was employed (see “Methods” for details). A current was injected into the ring-shaped electrode for operating the fabricated device as a polarization rotator. Since a magnetic field was generated around this electrode, no external coils or bulky magnets were required to induce the magneto-optical effect.

**Figure 2:**
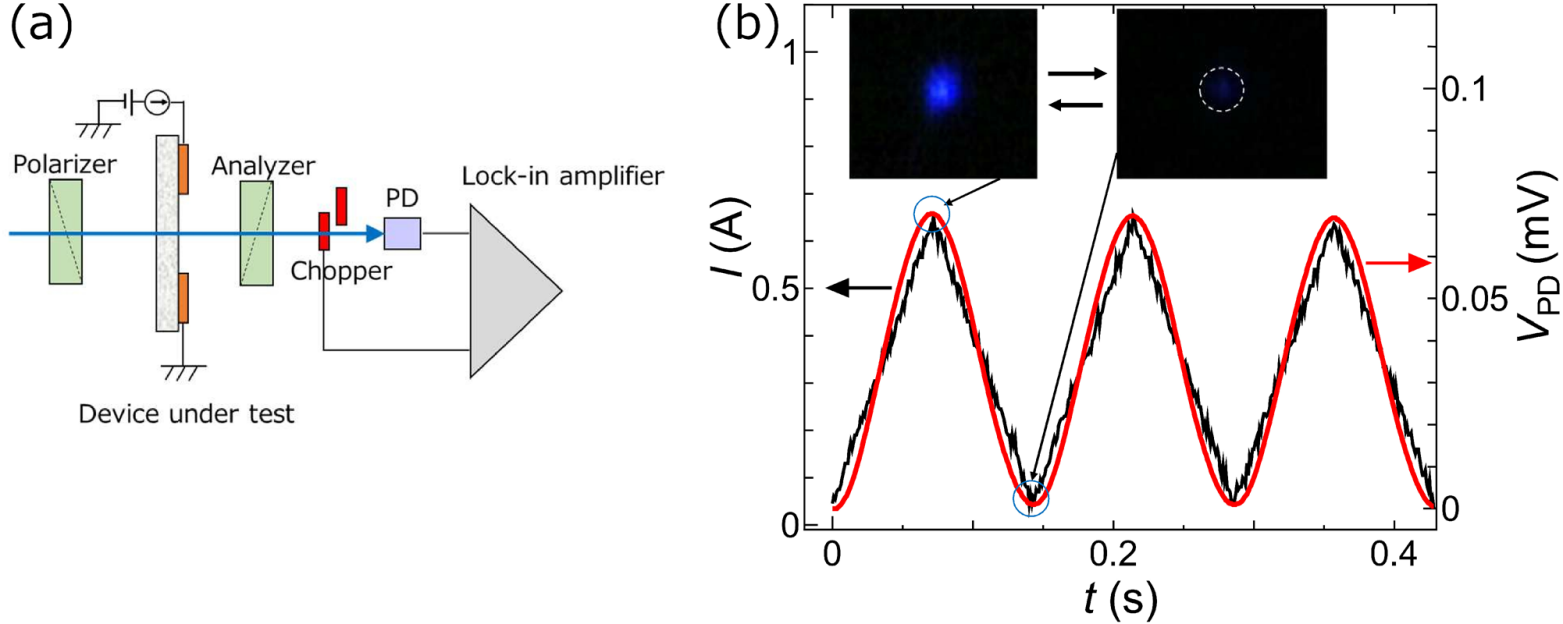
Evaluation of the polarization rotation characteristics. (**a**) Experimental setup. (**b**) Measured values of the temporal variation of the transmitted light intensity (V_PD_: red curve). Black curve is that of the triangular current (*I*) injected to the ring-shaped electrode, whose frequency and amplitude were 7 Hz and 600 mA, respectively. The *inset* in this figure shows snapshots of the transmitted light spot.

The polarization rotation characteristics were evaluated for linearly polarized 450 nm-wavelength laser light that was normally incident on the device surface. A commonly used measurement system was employed for evaluation, as is schematically illustrated in Fig. 2a. Briefly, linearly polarized light with a spot diameter of 100 µm was incident on the center of the ring-shaped electrode. In order to exclude additional rotation of the polarization due to birefringence caused by the optical anisotropy of the 4H-SiC crystal, the propagation direction of the incident light was precisely adjusted to be normal to the crystal surface and also to be parallel to the C-axis of the 4H-SiC crystal.

A polarization selector was constructed used the crossed Nichol configuration by using a polarizer (prism beam splitter (PBS) with an extinction ratio of 1 × 10^–3^) (Thorlabs: CCM1-PBS251/M) and an analyzer (polarizing plate) (Sigmakoki: SPF-30C-32). The device under evaluation was installed between them. The intensity of the light transmitted through the analyzer was measured by using a photodiode (Hamamatsu Photonics: S1226-18BK). For measuring with sufficiently high signal-to-noise ratio, a lock-in detection method was employed by using an optical chopper (frequency: 1.7 kHz) and a lock-in amplifier (Stanford Research Systems: SR830, time constant: 10 ms).

The red curve in Fig. 2b represents the measured values of the temporal variation of the transmitted light intensity. The black curve is that of the triangular current injected into the ring-shaped electrode. Its repetition frequency and amplitude were 7 Hz and 600 mA, respectively. The *inset* figures show snapshots of the transmitted light spot, which were taken by focusing and projecting the light beam on the screen. Polarization rotation can be clearly confirmed by comparing the images on the left and right in this inset, which were acquired at the top and bottom of the triangular current in this figure, respectively. The red curve shows that the transmitted light intensity varied by following the current variation, from which the polarization rotation angle of the transmitted light was quantitatively evaluated.

## Discussion

**Figure 3:**
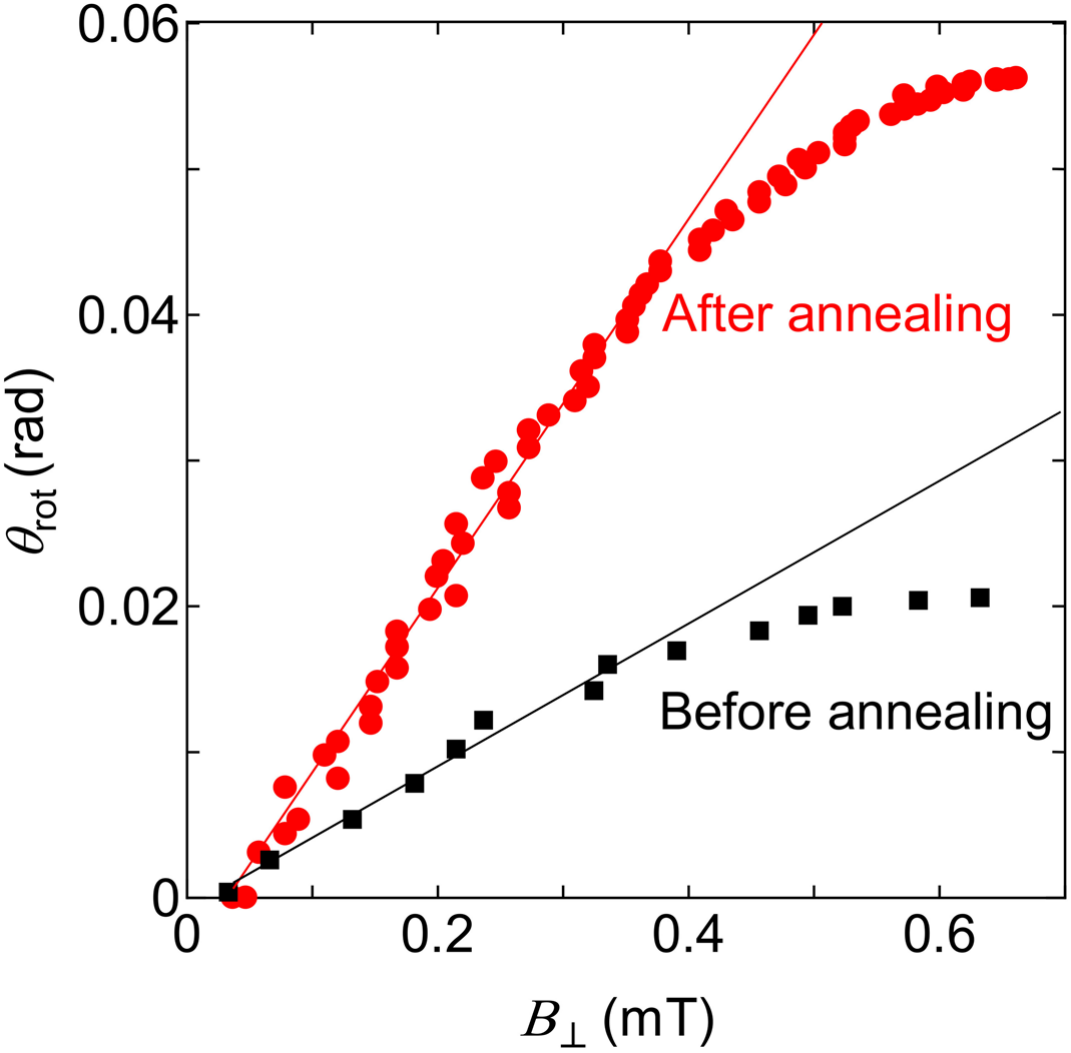
Measured relation between the magnetic flux density ($B_{ \bot }$) and the polarization rotation angle ($\theta_{{{\text{rot}}}}$). Closed red circles and black squares in the figure represent the device after and before DPP-assisted annealing, respectively. Solid lines were fitted to the measured values at $B_{ \bot }$ < 0.3 mT.

Figure 3 shows the relation between the magnetic flux density ($B_{ \bot }$) and the polarization rotation angle ($\theta_{{{\text{rot}}}}$), which was estimated from V_PD_ of Fig. 2b using the standard method for polarization measurement: Before injecting the current to the device, the analyzer was rotated and the rotation angle vs V_PD_ was measured., i.e., by referring to the relation between the rotation angle vs V_PD_ measured by rotating the analyzer.

**Figure 4:**
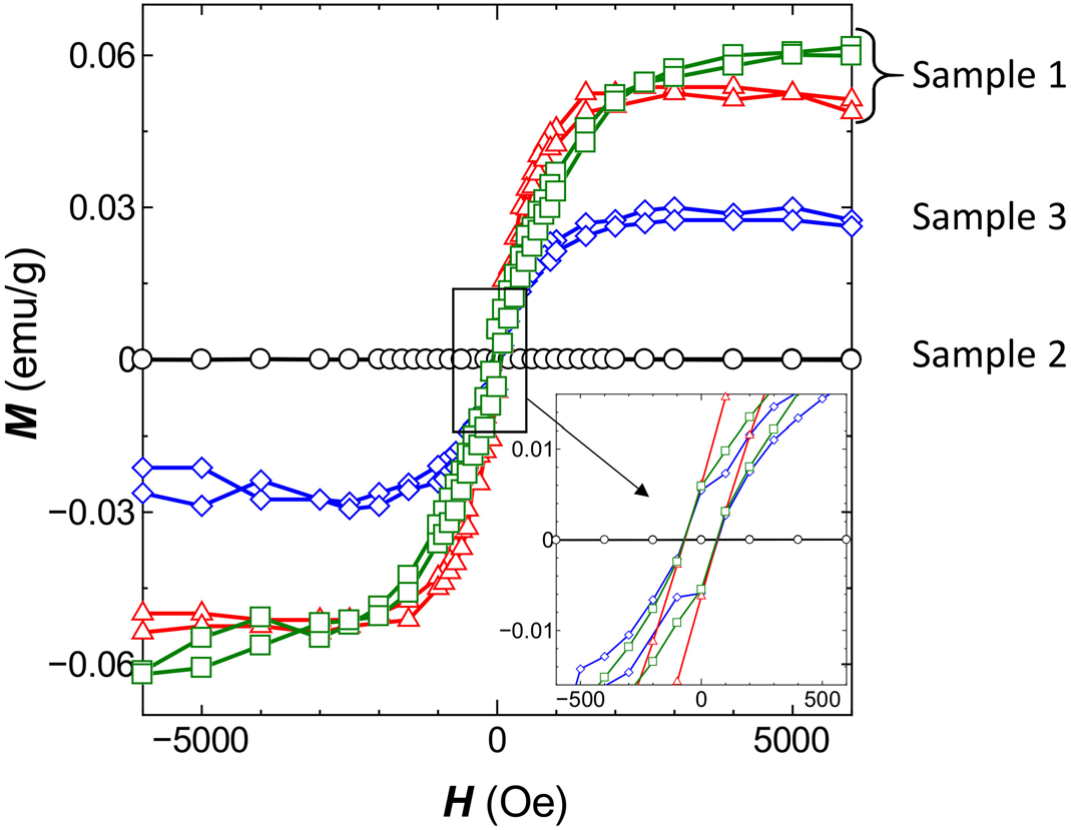
Relations between the applied magnetic field ***H*** (Oe) and the magnetization ***M*** (emu/g) at 300 K. Red triangles and green squares (Sample 1): A SiC crystal after DPP-assisted annealing with doping Al atoms, where the annealing times were 0.5 h and 72 h, respectively. Black circles (Sample 2): A SiC crystal without Al doping (no DPP-assisted annealing). Blue diamonds (Sample 3): A SiC crystal with Al doping (no DPP-assisted annealing). The *inset* shows the magnified relations near the origin of the graph.

The red circles and black squares represent the relation acquired after and before DPP-assisted annealing, respectively. The reason for the polarization rotation even before the DPP-assisted annealing will be given later by referring to Fig. 4. The value of $B_{ \bot }$ represents the vertical component of the magnetic flux density on the device surface, which was generated by the current injected into the ring-shaped electrode. Its value at the position of the transmitted light spot was evaluated to be 1.1 mT/A using a formula $B_{ \bot } = \mu_{0} I/d$, [where *d* is the ring diameter (1.1 mm)] derived from the Biot–Savart law. Also, from the slopes of the solid lines fitted to the measured values at $B_{ \bot }$ < 0.3 mT, the changes of the polarization angle per unit change of $B_{ \bot }$ were evaluated to be 5.15 × 10^–2^ rad/mT and 1.09 × 10^–1^ rad/mT before and after DPP-assisted annealing, respectively. These values represent the magneto-optic sensitivity $S$ represented in the unit of (rad/mT). The Verdet constant $V$ was derived from the relation $V = S \times ({\text{d}}B_{ \bot } /{\text{d}}I)$ and expressed in the unit (rad/A). Here, ${\text{d}}B_{ \bot } /{\text{d}}I$ (= 1.1 mT/A) is the value of the vertical component of the magnetic flux density that was generated by 1-A current injected into the electrode. In order to change the unit (rad/A) of $V$ to (rad/T.m), a common conversion formula 1 (A/m) = 1.26 × 10^–6^ (T) was used. As a result, The Verdet constant was expressed as $V\,$= 8.73 × 10^5^ $S$ (rad/T.m). By using this formula, the values of the Verdet constant, before and after the DPP-assisted annealing, were derived as 4.49 × 10^4^ rad/T.m and 9.51 × 10^4^ rad/T.m, respectively.

The result showed a two-times increase in this constant, which originated from the increased contribution from the DPP due to the DPP-assisted annealing. The saturation of $\theta_{{{\text{rot}}}}$ seen at $B_{ \bot }$ > 0.4 mT in Fig. 3 was attributed to the temperature increases in the device due to the Joule-heat generated by current injection. As an evicence, it was estimated experimentally and by the thermal analysis simulation that crystal temperature increased up to 300 °C by 1 A current injection. The saturation in Fig. 3 was not due to the deterioration of the SiC crystal quality but to the decreased efficiency of generating the magnetic field (${\text{d}}B_{ \bot } /{\text{d}}I$) by the current injected into the ring electrode. The reason for this is that, with the increase in temperature, a part of the current in the ring electrode leaked out to the SiC crystal due to the increases and decreases of the resistance of the metallic wire used for the ring electrode and that of the semiconductor SiC crystal, respectively. Thus, a part of the current in the ring electrode leaked out to the SiC crystal, resulting in a decrease in ${\text{d}}B_{ \bot } /{\text{d}}I$. However, the performance of the device was evaluated in the region $B_{ \bot } \,$< 0.3 mT, where it was free from the effects of temperature increases; i.e., the value of ${\text{d}}B_{ \bot } /{\text{d}}I$ was maintained constant. For comparison, the Verdet constants of TGG, CeF_3_, and PrF_3_, which are the materials used for conventional optical isolators in the visible range, are on the order of 10^2^ rad/T.m^23^. The above value of 9.51 × 10^4^ rad/T.m is 10^2^-times higher than this, by which a gigantic magneto-optical effect was confirmed. Furthermore, the above value is as high as that of the typical ferromagnetic YIG (10^5^–10^6^ rad/T.m) in the visible range^4^.

In order to examine the origin of such a large polarization rotation capability realized by using the indirect-transition-type semiconductor SiC crystal, magnetization curves of a SiC single crystal (5 mm × 5 mm × 0.3 mm) after the DPP-assisted annealing (Sample 1) were acquired at a temperature of 300 K by using a SQUID (Quantum Design: MPMS with EverCool). For comparison, the magnetization curves were acquired also for a SiC crystal without Al doping (Sample 2) and for a SiC crystal doped with Al doping before DPP-assisted annealing (Sample 3). From preliminary measurements, it was confirmed that the values of the magnetization per unit mass* M* (emu/g) showed diamagnetic characteristics, that is, a linear dependence on the applied magnetic field ***H*** (Oe), in the range of − 10 kOe ≦ ***H*** ≦ 10 kOe. This dependence is reasonable because the SiC single crystal is a semiconductor.

For a more detailed discussion, Fig. 4 shows the magnified relation between ***H*** and ***M*** obtained by subtracting the contributions of the diamagnetic component from the measured values of ***M***. The curves in this figure have several typical characteristics: The values of ***M*** of Sample 1 (green squares and red triangles) are remarkably larger than those of other samples. Those of Sample 2 (black circles) are negligibly small. Sample 3 (blue diamonds) exhibit a certain amount of ***M***. The curves for Samples 1 and 3 show hysteresis characteristics. Furthermore, they exhibit a coercive force, as shown in the *inset* of Fig. 4. These characteristics indicate that the ferromagnetic characteristics were induced by doping Al atoms.

The saturated value of* M* of Sample 1 (green squares and red triangles) is about twice that of Sample 3. This figure also indicates that the saturated value increased with increasing DPP-assisted annealing time. This is the evidence that the large MO effect, realized by doping Al, was boosted by a factor of two by the DPP-assisted annealing. Thus, the fabricated device exhibited a larger MO effect than that exhibited by conventional materials, and this led to the large poralization-rotation of the transmitted visible light. It has been pointed out, based on a thermodynamic model for the two-level systems^21,24^, that the Al dopants are apt to gradually diffuse in time due to the Joule energy generated by the current injected for operating the device. As a result, the magnitude of the MO effect, and thus the polarization-rotation angle, were apt to decrease gradually in time during the device operation. However, the DPP-assisted annealing could suppress this diffusion by forming an irreversible potential barrier to the Al dopants. As a result, even during the device operation, the spatial distribution of the Al dopants maintained the original profile that was formed by the DPP-assisted annealing. This indicates that the DPP-assisted annealing was indispensable in stabilizing the device operation.

As a reference, Song et al*.* have reported some ferromagnetic characteristics in SiC, which were attributed to doped Al atoms^25^. Although their specimens correspond to Sample 3 in the present study before the DPP-assisted annealing, they were prepared by sintering powdered silicon, carbon, and aluminum. Thus, that material is not suitable for use as a homogeneous and transparent material for the present polarization rotator in the visible range. In the present study, by employing DPP-assisted annealing, conspicuous ferromagnetic characteristics were induced in Sample 1 to realize a larger polarization rotation. The origins of such conspicuous characteristics are as follows: First, in Sample 3, the implanted Al atoms having a random spatial distribution form dimers (Al atom pairs), and the parallel spins in the pairs induce ferromagnetic characteristics. Second, in Sample 1, the spatial distribution of Al atoms was controlled autonomously by the DPP-assisted annealing, resulting in an increase in the number of Al atom pairs. Since it has been found that the triplet state of the electron orbital in an Al atom pair is more stable than the singlet state^26,27^, the parallel spins induced more significant ferromagnetic characteristics^28^. Detailed discussions on these origins are currently underway.

## Conclusion

This paper reported the fabrication and operation of a transmission-type polarization rotator for visible light with a wavelength of 450 nm using indirect-transition-type semiconductor crystalline SiC to which Al atoms were implanted as a p-type dopant. A novel DPP-assisted annealing method was used for fabrication. The fabricated device was much more compact than the conventional optical isolators because the optical path length required for the polarization rotation in the present device was as short as the thickness of the p–n junction. Also, for operating the fabricated device, no external coils or bulky magnets were required to apply a strong magnetic field to the device. A magnetic flux density as low as mT was sufficient, and this was generated by injecting current into a ring-shaped electrode on the device surface. The fabricated device exhibited a gigantic magneto-optical effect. Specifically, the Verdet constant was as large as 9.51 × 10^4^ rad/T.m at a wavelength of 450 nm, which was 10^2^-times higher that of TGG and as high as that of the typical ferromagnetic YIG in the visible range. Furthermore, SQUID measurements confirmed that the SiC crystal exhibited conspicuous ferromagnetic characteristics as a result of the DPP-assisted annealing. It is expected that this device can be used as an efficient transmission-type light modulator or an optical isolator for future information processing systems.

## Methods

This section describes the process of fabricating the SiC device. For substrate preparation, first, an n-type 4H-SiC single-crystal was used, whose surface orientation was (0001). The n-type dopant (N atoms) density was 1 × 10^18^ cm^−3^. Second, a 10 µm-thick n-type epitaxial layer (n-type dopant (N atoms) density 1 × 10^16^ cm^−3^) was deposited on the crystal. Finally, in order to form a p–n junction, Al atoms serving as a p-type dopant were implanted into the (0001) surface by three-step ion implantation with acceleration energies of 700 keV, 350 keV, and 15 keV. The peak concentration was 1 × 10^19^ cm^−3^. It was confirmed by preliminary experiments that the absorption coefficient of the SiC substrate was about 70 cm^−1^ at a wavelength of 450 nm. That is, an optical transmittance of the formed p–n homojunction (thickness: about 1 µm or less) was estimated to be as high as 99.3% at a wavelength of 450 nm. After ion-implantation of the Al dopants, post-implantation annealing was employed for crystallinity recovery. Specifically, the SiC crystal was annealed in a high-temperature furnace at 1,800 °C for five minutes. This was repeated two times. Since the highest temperature increase (300 °C) induced by the Joule energy during DPP-assisted annealing was much lower than 1,800 °C, its effect on further improvement of the crystal quality, and thus on the polarization rotation, was negligible.

After electrodes were formed on the top and bottom surfaces of the SiC substrate, the substrate was diced to form a device with dimensions of 3 mm × 3 mm. The ring-shaped p-electrode on the top surface was formed by a Cr/Au (100 nm/700 nm thick) film whose diameter and line width were 1.1 mm and 0.1 mm, respectively. The planar n-electrode on the bottom surface was a Cr/Pt/Au (30 nm/200 nm/700 nm thick) film with a 2 mm-diameter aperture at the center for allowing the incident light to pass through the device. These two electrodes were used for current injection to the p–n junction in the process of the DPP-assisted annealing. No ferromagnetic metals were used for these electrode materials, and it was confirmed that the metals used for the electrodes did not make any contribution to the magneto-optical effect exhibited by this device. For reference, GD-MS analysis (Nu Instruments: ASTRUM) confirmed that the concentration of the ferromagnetic metal impurity in the SiC crystal was as low as 0.036 ppm.

DPP-assisted annealing was carried out on the diced device: A forward bias voltage of 19 V (current density 0.022 A/mm^2^) was applied for Joule heating. The top surface of the device was simultaneously irradiated with laser light (typical irradiation time is given in the caption for Fig. 4). The optical power and wavelength were 20 mW and 405 nm, respectively. By momentum exchange between the phonons in the DPP and the electrons in the conduction band, and electron–hole recombination, photon emission was realized, converting a part of the Joule energy to optical energy. Since the emitted photons propagated out from the device and dissipated, the diffusion rate of Al atoms was locally decreased and, as a result, the spatial distribution of Al atoms was controlled autonomously. This autonomous control played an essential role in the DPP-assisted annealing.

The device fabricated by this process operated as a polarization rotator by injecting current only to the ring-shaped p-electrode, while the planar electrode on the bottom surface was not used. It exhibited a gigantic magneto-optical effect in the visible range (wavelength, 450 nm) even though the SiC crystal was an indirect-transition-type semiconductor.
